# Supplementary material for: Poly(carboxylated ether)s as Cement Additives: The Effect of the Addition Method on Hydration Kinetics
Source: Materials (Basel). 2024 Oct 31;17(21):5343. doi: 10.3390/ma17215343 (PMC11547811; doi:10.3390/ma17215343)
Supplement: Supplementary file 1 [file materials-17-05343-s001.zip › materials-3242610-supplementary.pdf]

Supporting Information for

Poly(carboxylated ether)s as Cement Additives: The Effect of the Addition Method on Hydration Kinetics

Sara Beldarrain [1], Guido Goracci [2], Jorge S. Dolado [2], Aitor Barquero [1,] and Jose Ramon Leiza [1,]**

[1]POLYMAT, Kimika Aplikatua Saila, Kimika Fakultatea, University of the Basque Country, UPV/EHU, Joxe Mari Korta Zentroa, Tolosa Hiribidea 72, 20018 Donostia/San Sebastián, Spain.

[2]Centro de Física de Materiales (CSIC, UPV/EHU) Materials Physics Center (MPC) Paseo Manuel de Lardizabal 5, 20018 Donostia/San Sebastián, Spain.

** Corresponding authors*

Aitor Barquero. aitor.barquero@ehu.eus

Jose Ramon Leiza. jrleiza@ehu.eus

Section S1. Synthesis and characterization of PCEs.

The PCEs were synthesized by semibatch aqueous solution copolymerization in a 250 mL glass jacketed reactor with a thermostatic water bath and a mechanical turbine stirrer at 200 rpm. The reactor was equipped with a feeding inlet, a condenser, nitrogen bubbling and a sampling device. Deionized water (100 g) was loaded to the reactor and heated to 80 °C. During the heating, the system was purged using a nitrogen flow at 15 mL·min^{-1} and maintained during the reaction. When the reaction temperature (80 °C) was achieved, the feeding started. The monomers (MAA and PEGMA), initiator (KPS) and sodium bicarbonate dissolved in the rest of the water were fed in the same stream for three hours at 0.56 g·min-1. After, post polymerization was carried out for an hour, with the aim of reaching full conversion of the monomer. The solids content (SC) and amount of chain transfer agent (CTA) varied depending on the reaction in order to control viscosity during the reaction.

The PCEs were characterized by determining the individual conversion of each monomer by 1H-NMR, the monomer sequence distribution by a Gaussian/Lorentzian deconvolution of the carbonyl peaks of MAA in ^{13}C-NMR, and the molar mass distribution of the polymers was analysed by Asymmetric-Flow Field-Flow Fractionation with Multi Angle Light Scattering and

Refractive Index detectors (AF4/MALS/RI). More details can be found in a previously published article (1).

Section S2. Mineralogical composition and specific surface area of the CEM type I 52.2R OPC.

The cement used was CEM type I 52.2R Ordinary Portland Cement (OPC), which was kindly supplied by Lemona Cements S.A. Its mineralogical composition, determined by X-ray diffraction (BRUKER D8 Advance) using Rietveld refinement, is shown in Table SI.1. Its specific surface area calculated according to Blaine fineness method (UNE EN 196-6:2010) was 0.4523 $m^2 \cdot g^{-1}$.

Table S1. *Mineralogical composition of the Ordinary Portland Cement type I 52.5R obtained by XRD and Rietveld refinement.*

Rietveld Analysis

**Phases**	**wt %**
Amorphous	10.8
C_3S	48.1
β-C_2S	17
Cubic C_3A	6.8
C_4AF	5.9
Calcite	6.1
Akermanite	2.1
Portlandite	1
Gypsum	0.9
Quartz	0.6
Bassanite	0.6
Dolomite	-

Section S3. Hydration kinetics

Figure S1. kinetics of direct and delayed addition of L series.

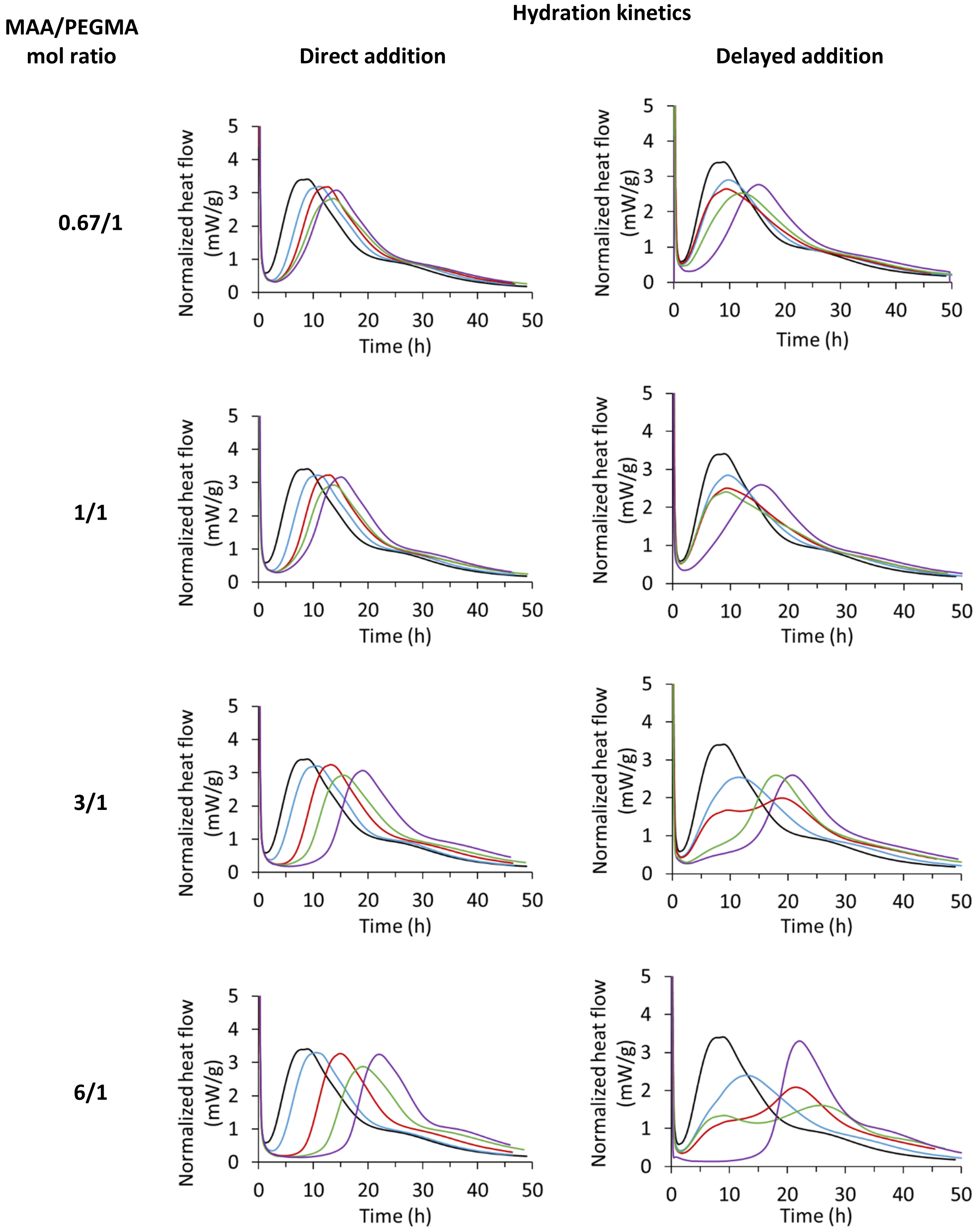

Figure S1. Released heat of hydration over time for PCEs of series L and different MAA/PEGMA molar ratio (0.67/1, 1/1, 3/1, and 6/1) at different PCE dosages (blue – 1 mg_{PCE}/g_{OPC}; red – 2 mg_{PCE}/g_{OPC}; green – 3 mg_{PCE}/g_{OPC}; purple – 4 mg_{PCE}/g_{OPC}). The black line belongs to the reference sample where no PCE was added. Results on direct addition were reprinted with permission of reference S. Beldarrain et al. Rationalizing the Effect of the MAA/PEGMA Ratio of Comb-

Figure S2. Total heat of hydration over time.

0.67/1 M

1 mg$_{PCE}$/g$_{OPC}$

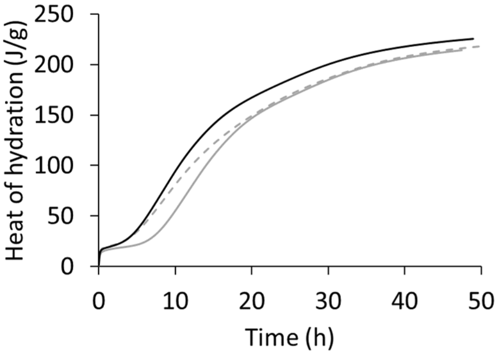

2 mg$_{PCE}$/g$_{OPC}$

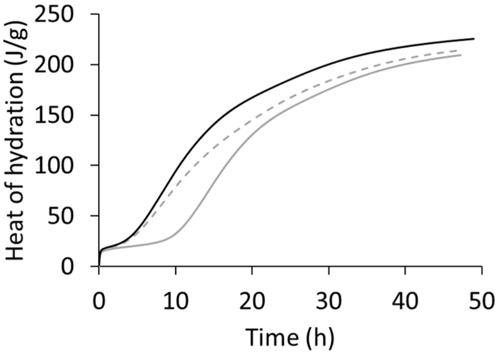

3 mg$_{PCE}$/g$_{OPC}$

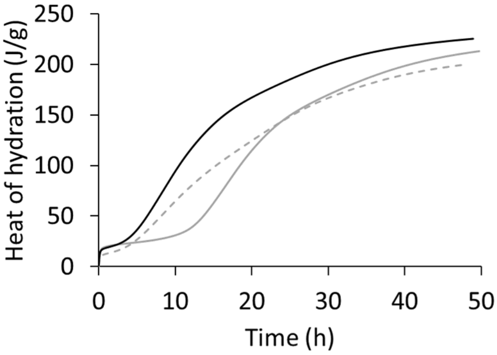

4 mg$_{PCE}$/g$_{OPC}$

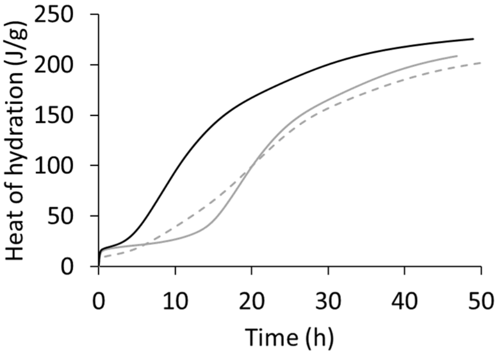

1/1 M

1 mg$_{PCE}$/g$_{OPC}$

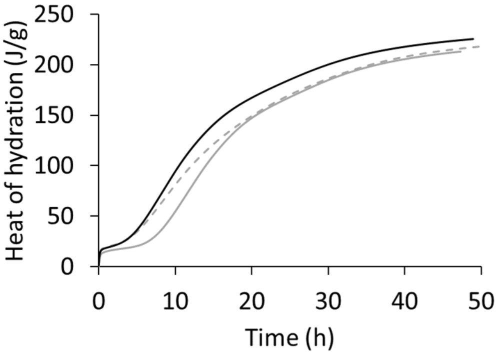

2 mg$_{PCE}$/g$_{OPC}$

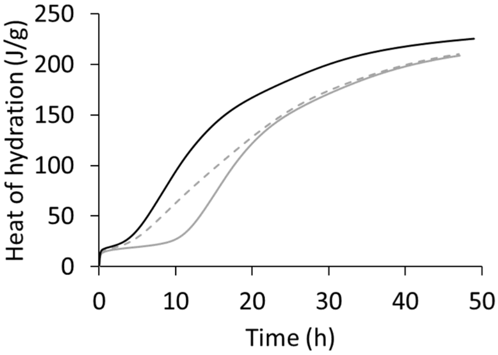

3 mg_{PCE}/g_{OPC}

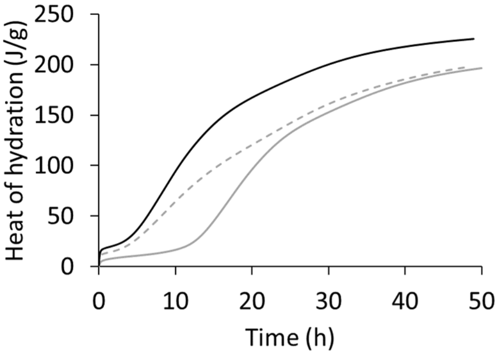

4 mg_{PCE}/g_{OPC}

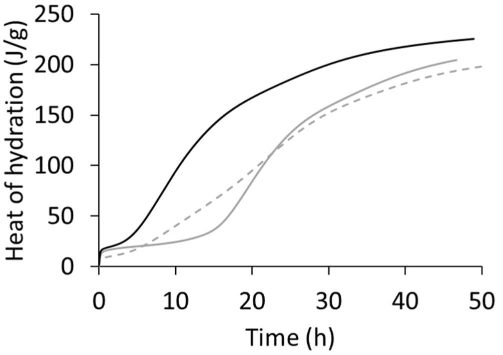

6/1 M

1 mg_{PCE}/g_{OPC}

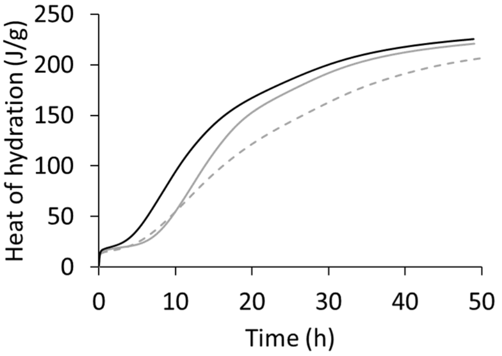

2 mg_{PCE}/g_{OPC}

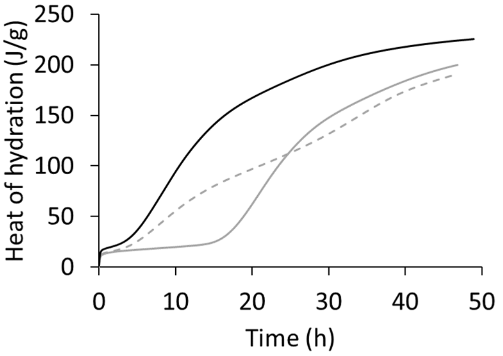

3 mg_{PCE}/g_{OPC}

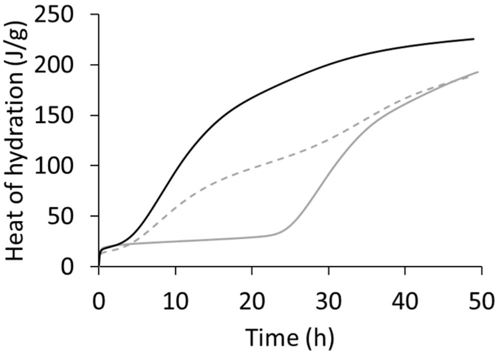

4 mg_{PCE}/g_{OPC}

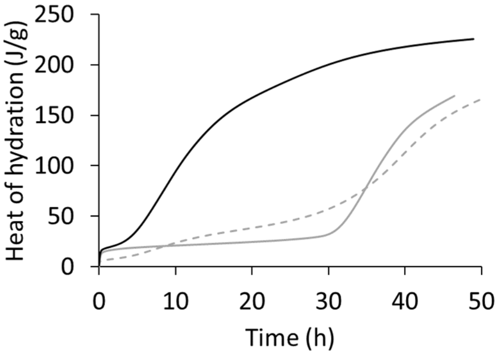

0.67/1 L

1 mg_{PCE}/g_{OPC}

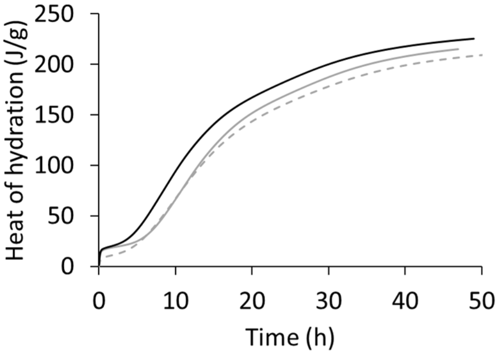

2 mg_{PCE}/g_{OPC}

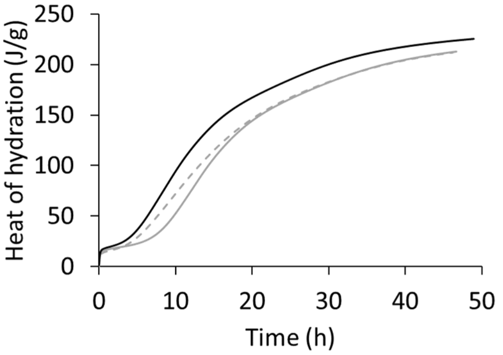

3 mg_{PCE}/g_{OPC}

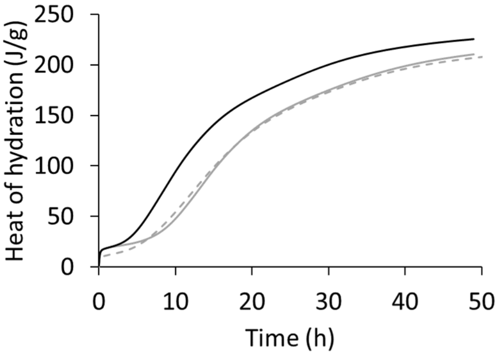

4 mg_{PCE}/g_{OPC}

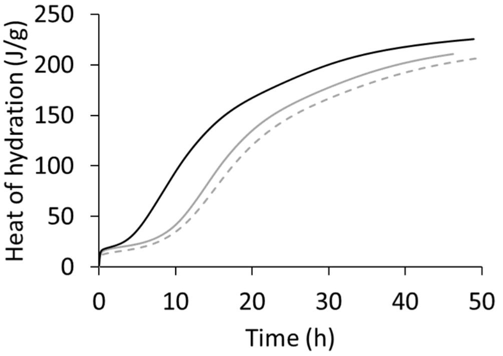

1/1 L

1 mg_{PCE}/g_{OPC}

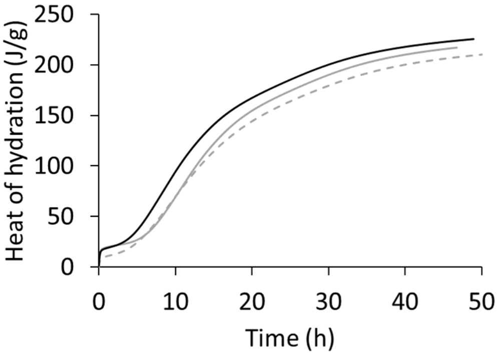

2 mg_{PCE}/g_{OPC}

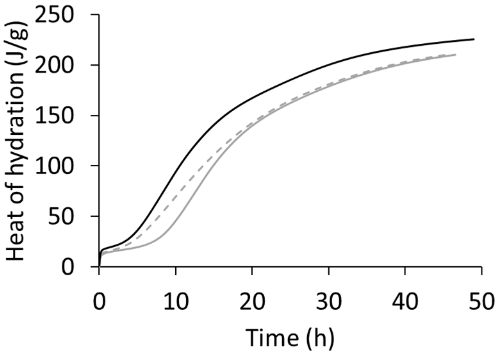

3 mg_{PCE}/g_{OPC}

4 mg_{PCE}/g_{OPC}

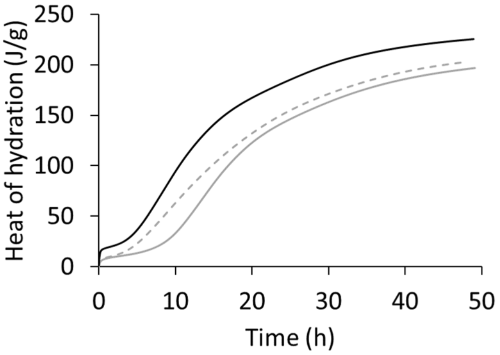

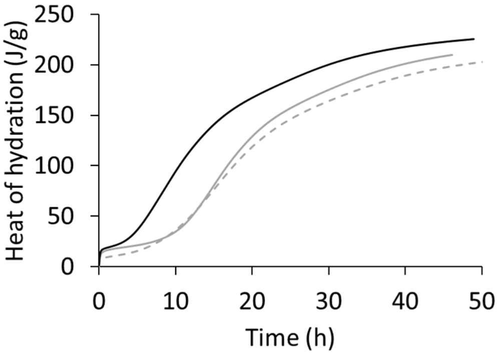

3/1 L

1 mg$_{PCE}$/g$_{OPC}$

2 mg$_{PCE}$/g$_{OPC}$

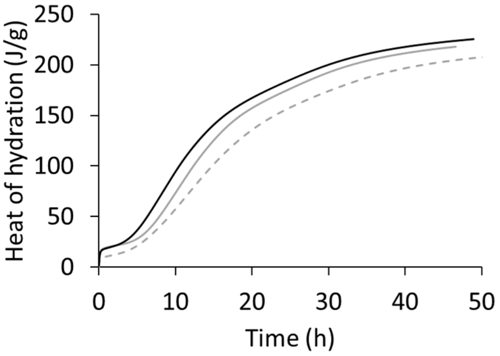

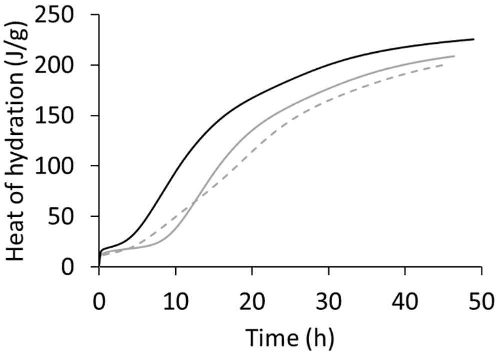

3 mg$_{PCE}$/g$_{OPC}$

4 mg$_{PCE}$/g$_{OPC}$

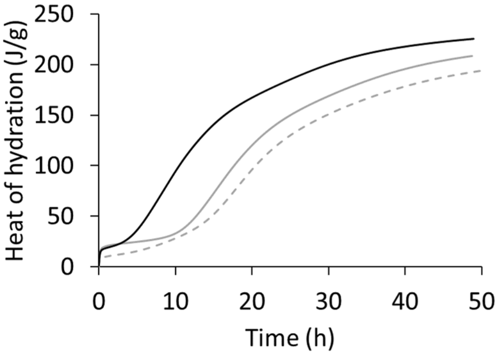

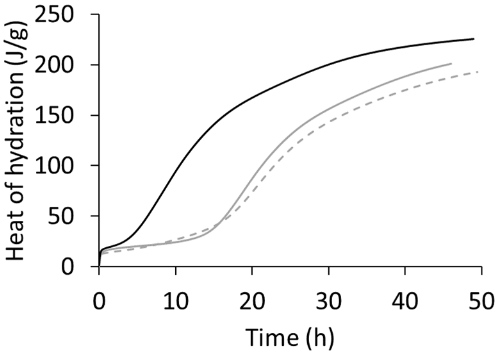

6/1 L

1 mg$_{PCE}$/g$_{OPC}$

2 mg$_{PCE}$/g$_{OPC}$

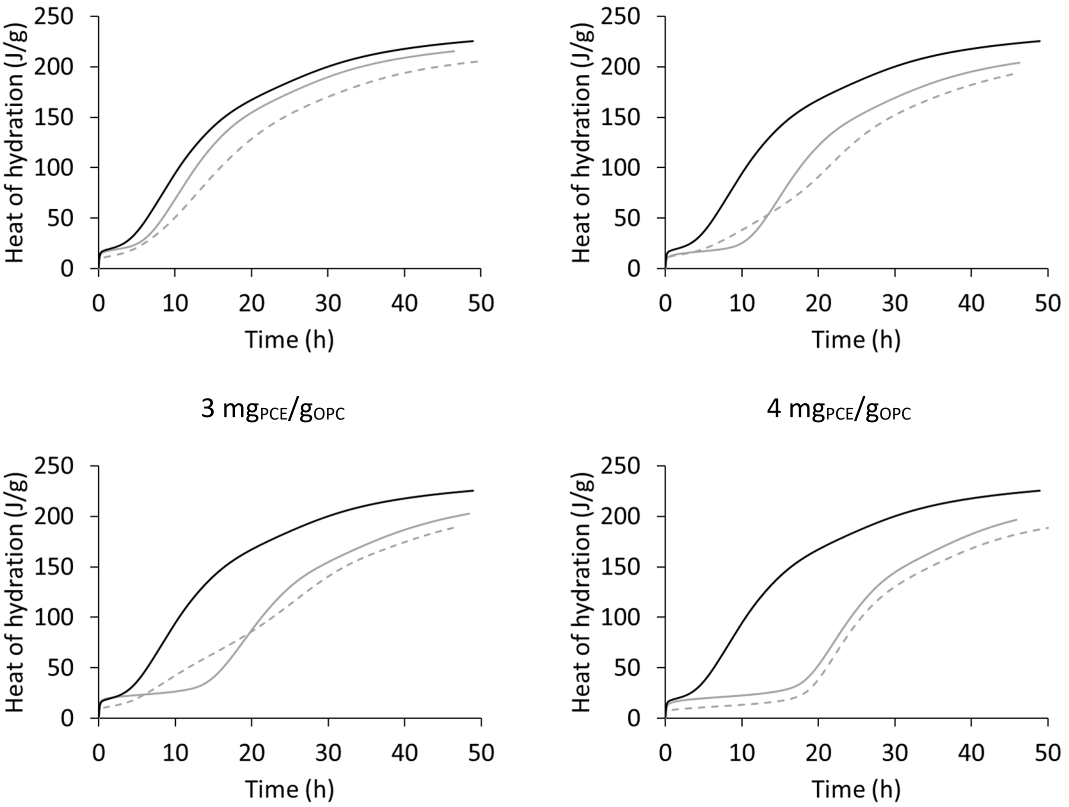

Figure S2. *Total heat of hydration over time for the PCE of series L at different MAA/PEGMA molar ratios added to the system through direct (continuous grey line) and delayed addition (dashed grey line). Black line belongs to the reference sample, to which no PCE was added.*
